# Skin Sodium and Hypertension: a Paradigm Shift?

**DOI:** 10.1007/s11906-018-0892-9

**Published:** 2018-09-13

**Authors:** Viknesh Selvarajah, Kathleen Connolly, Carmel McEniery, Ian Wilkinson

**Affiliations:** Division of Experimental Medicine and Immunotherapeutics, University of Cambridge, Box 98, Addenbrookes Hospital, Cambridge, CB2 0QQ UK

**Keywords:** Blood pressure, Salt, Skin, Sodium, VEGF-C

## Abstract

**Purpose of Review:**

Dietary sodium is an important trigger for hypertension and humans show a heterogeneous blood pressure response to salt intake. The precise mechanisms for this have not been fully explained although renal sodium handling has traditionally been considered to play a central role.

**Recent Findings:**

Animal studies have shown that dietary salt loading results in non-osmotic sodium accumulation via glycosaminoglycans and lymphangiogenesis in skin mediated by vascular endothelial growth factor-C, both processes attenuating the rise in BP. Studies in humans have shown that skin could be a buffer for sodium and that skin sodium could be a marker of hypertension and salt sensitivity.

**Summary:**

Skin sodium storage could represent an additional system influencing the response to salt load and blood pressure in humans.

## Introduction

Chronically elevated blood pressure, known as hypertension, represents an imminent global health challenge. Hypertension is responsible for over 10 million deaths annually and is one of the foremost modifiable risk factors for stroke, heart failure, ischaemic heart disease and chronic kidney disease [1–3]. Hypertension currently affects nearly one third of the population, and its prevalence is increasing worldwide [4]. Despite this pervasiveness, the precise origins of hypertension remain unclear, with research primarily focused on the kidney, brain, heart and blood vessels. Large population studies suggest that excessive dietary sodium, principally as the chloride salt, is an important trigger for hypertension, with the kidney considered to be the main organ regulating the haemodynamic response to salt intake [5, 6]. In this review, we examine emerging evidence supporting the role of the skin in sodium homeostasis and the regulation of blood pressure and novel extrarenal mechanisms involved in these.

## Rethinking the Mechanisms for Salt Sensitivity of Blood Pressure (SSBP)

Salt sensitivity of blood pressure (SSBP) refers to the physiological trait by which BP of certain individuals exhibits changes parallel to changes in salt intake, while individuals without this trait are termed salt resistant [7]. SSBP is more common with greater age, Afro-Caribbean descent and individuals with hypertension, diabetes mellitus and chronic kidney disease. The variability in this trait and the mechanisms by which sodium influences blood pressure are not fully understood [7, 8]. In the classical Guytonian model, sodium intake in excess of renal excretory capacity causes an osmotically driven expansion of the extracellular fluid volume. This leads to an increase in plasma volume, venous return, and cardiac output, which in turn produce a rise in systemic blood pressure [9]. Thus, in this traditional paradigm, a defect in renal sodium excretion is the basis for salt sensitivity; conversely, salt-resistant individuals are protected from salt-induced BP rises because they can rapidly excrete a salt load without retaining sodium [10]. Others have expanded on this model, suggesting that SSBP occurs with a subnormal decrease in renal and peripheral vascular resistance in response to a high salt intake, rather than an increase in sodium retention and cardiac output, with the kidney maintaining a central role [11].

More recent studies have similarly challenged this view in humans. Schmidlin and Laffer et al. noted that both salt-resistant and salt-sensitive normotensive individuals underwent similar degrees of body sodium retention with acute dietary salt loading, demonstrating that in salt-resistant individuals, sodium retention occurred without adverse effects on BP [12, 13, 14••].These studies revealed that salt-resistant individuals can adapt to a salt load via vasodilation concomitant to increased cardiac output, while this vasodilatory response is attenuated those who are in salt sensitive [12, 13, 14••]. In general, these observations refute the view that salt sensitivity is solely due to deficiencies in renal excretion of sodium.

## A ‘Three-Compartment Model’ of Body Sodium

Total body water (TBW) has historically been divided into two compartments, the intracellular fluid (ICF) and the extracellular fluid (ECF), with this latter compartment comprising the intravascular and interstitial spaces. Total body sodium (TBNa) has been similarly compartmentalised [15].While intracellular sodium and fluid volume are tightly regulated to protect cells against detrimental volume changes, the intravascular and interstitial spaces are generally believed to be in equilibrium.

Following an increase in dietary salt intake, sodium accumulates in the extracellular space. In theory, each 140 mmol of additional sodium must be coupled with the accumulation of ~ 1 l of water in the extracellular fluid to maintain osmolality. However, four carefully conducted longer-term sodium balance studies in healthy humans have shown that large amounts of sodium can accumulate without commensurate water [16–19]. Of these studies, the Mars 500 study, which investigated sodium metabolism at constant salt intake under controlled conditions for either 105 or 250 days, showed that sodium is rhythmically stored and released independent of salt intake, and that BP, body weight and extracellular water were not coupled to urine sodium excretion as expected [19]. A further study, which assessed sodium and water excretion in healthy humans after infusion with hypertonic saline, found that sodium recovery in the urine was only half of the expected amount, indicating that some of the infused sodium was retained in an osmotically inactivated form [20••]. These observations support the existence of non-osmotic storage of excess sodium (sodium accumulation without commensurate water retention) in an additional third compartment, suggesting the existence of ‘extra-renal’ mechanisms for sodium homeostasis.

## The Skin as a ‘Third Compartment’ of Body Sodium and Relevance to BP

The skin is the largest organ in the human body, comprising 6% of body weight and forming a significant component of the interstitium [21, 22]. The skin consists of two tissue layers: the epidermis, the external layer consisting of non-stratified epithelial cells and the dermis, which consists mainly of connective tissue [22]. The epidermis is approximately 50–200 μm thick and acts as a physical barrier against microorganisms and water loss, while the dermis is relatively acellular, comprised of fibroblasts, blood vessels, lymphatics and nerves in an extracellular matrix of collagen, elastin and glycosaminoglycans [22, 23]. The skin is a rich source of nitric oxide, a major regulator of vascular tone, containing ten times the levels in the circulation [24]. Blood flow in the skin is dynamic, ranging from as low as 1% in cold temperatures to as high as 60% in erythroderma and heat stress [25, 26••]. These properties of the skin would suggest the potential for influencing systemic BP [25, 26••].

From as early as 1909, direct chemical measurements indicated that the skin is a depot for sodium, chloride and water, although the exact relevance of skin electrolytes was not known [27–30]. In 1978, Ivanova et al. showed that skin sodium in white rats increased with dietary salt loading and observed that this was associated with an increase in sulphated glycosaminoglycans [31]. Glycosaminoglycans (GAGs) are linear polysaccharide chains of variable length consisting of repeating disaccharide units [32, 33]. Due to the presence of carboxyl and sulphate functional groups on the disaccharide units, GAGs possess significant negative charge densities capable of facilitating the non-osmotic storage of sodium in the interstitium, as described in a recent comprehensive review [34••]. It should be noted that the skin is not the only place with high glycosaminoglycan content, and non-osmotic sodium storage can occur elsewhere in the interstitium.

In 2002, Titze et al. first proposed the connection between skin sodium, GAGs and BP following a series of rat experiments [35–39]. Work by this group revealed that GAG polymerisation facilitates osmotically inactive sodium storage in the skin, enabling skin sodium concentrations to rise as much as 180–190 mmol/l without commensurate increases in skin water content. This osmotically inactive sodium storage could therefore serve as a mechanism for buffering volume and blood pressure following changes in salt intake [36–39].

Titze et al. calculated that osmotically inactive sodium storage in salt-resistant rats was threefold higher than in salt-sensitive rats, based on body sodium and body water measurements [35]. They further demonstrated that male rats had a higher capacity for osmotically inactive skin sodium storage compared to fertile female rats, while ovariectomised rats had no capacity for osmotically inactive sodium accumulation [36]. The above findings led to the conclusion that the skin functions a ‘third compartment’ of body sodium, with a dynamic capacity for sodium storage and buffering volume and blood pressure changes with salt intake.

## Tissue Macrophages and the Lymphatics Influence Sodium Balance, Interstitial Volume and Blood Pressure

**Fig. 1:**
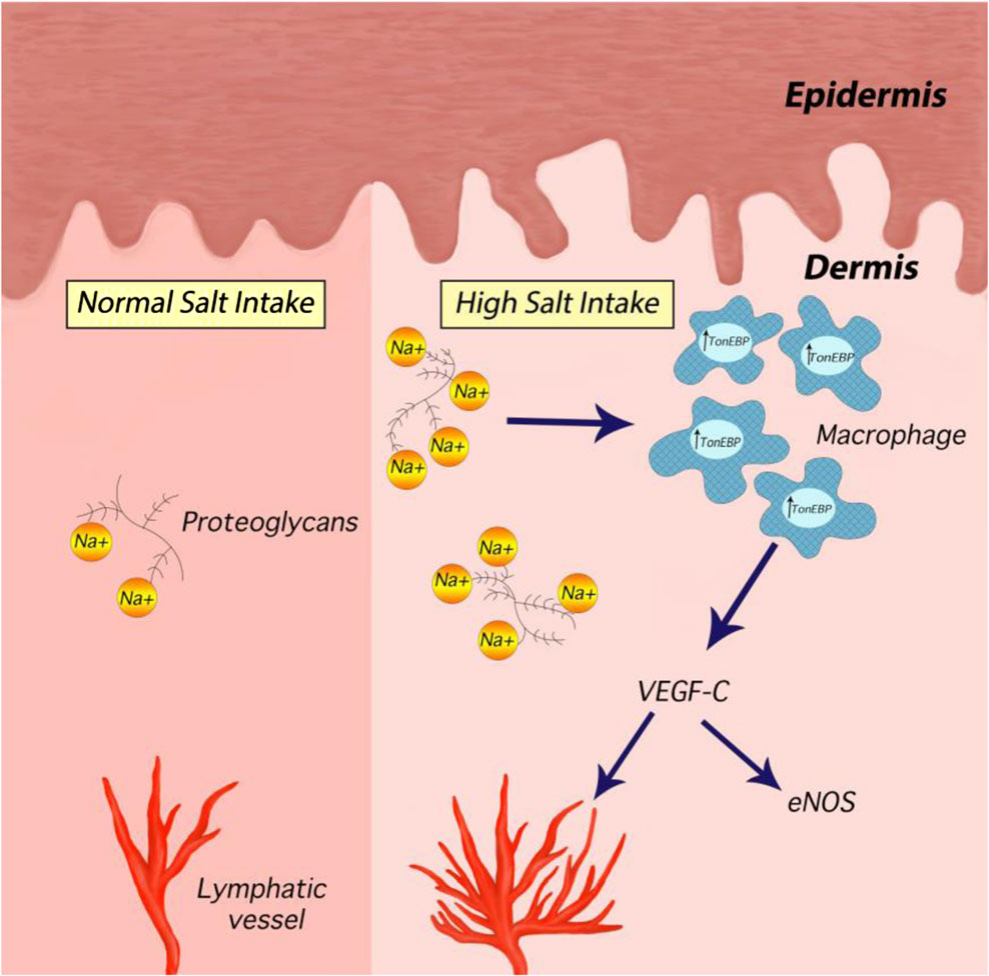
A novel extra-renal mechanism for buffering dietary salt. Under normal conditions, Na^+^ binds to negatively charged GAGs in the dermal interstitium, without commensurate water, allowing high concentrations of Na^+^ to accumulate in the skin. During salt loading, the Na^+^-binding capacity of GAGs is exceeded and interstitial hypertonicity develops. This leads to an influx of macrophages, which release an osmosensitive transcription factor (TonEBP). This induces the secretion of VEGF-C in an autocrine manner, leading to lymphangiogenesis. The enhanced lymphatic network increases Na^+^ transport back into the circulation, for eventual removal by the kidneys, preventing a blood pressure rise with salt loading (Adapted from Marvar et al. [42]. Illustrated by Gökçen Ackali)

In recent years, it has become apparent that cells of the innate and adaptive immune system play a role in hypertension and cardiovascular disease [40]. Work in rodent skin has showed that macrophages mediate an additional adaptive mechanism that functions during periods of high salt intake (Fig. 1) [38, 39, 41]. Following salt challenge, skin sodium concentration increased and the resultant hypertonicity caused recruitment of macrophages which activated tonicity-responsive enhancer binding protein (TonEBP). TonEBP increased the expression of vascular endothelial growth factor C (VEGF-C) gene via autocrine signalling. By mediating VEGF-C, the macrophage response restructured the interstitial lymphatic network, enabling drainage of water and electrolytes from the skin into the systemic circulation. VEGF-C also and induced expression of endothelial nitric oxide synthase (eNOS), causing vasodilation via nitric oxide (NO) production. These processes evidently serve to buffer the haemodynamic effects to salt loading [38]. VEGF-C and TonEBP antagonism and genetic deletion and consequent disruption of the above pathway caused salt sensitivity in these rodents [38, 39, 41].

In summary, macrophages exert a homeostatic function in the skin via TonEBP and VEGF-C, regulating clearance of osmotically inactive stored salt via cutaneous lymphatic vessels, buffering the haemodynamic response to dietary salt. In this process, the lymphatics serve as a connection between skin as a ‘third compartment’ of body sodium and the circulation and this appears to be mediated by VEGF-C.

## Recent Evidence for the Role of the Skin in Sodium Homeostasis and BP Regulation in Humans

Most of the novel mechanisms described above linking dietary salt, skin sodium, and BP were examined in rodent models. The rest of this review will focus on recent studies examining skin sodium in humans, its relevance to human sodium homeostasis and BP regulation and possible mechanisms linking these.

## Insights from Sodium MRI of the Skin in Humans

For over 30 years, the use of ^23^Na-MRI spectroscopy has enabled non-invasive in vivo assessment of sodium concentrations in human tissue [43]. Recently, researchers have begun using ^23^Na-MRI spectroscopy to measure sodium in calf skin and muscle alongside water measurements using traditional proton MRI [44, 45, 46••, 47, 48, 49••].

Initial experiments showed that skin sodium was positively correlated with age, and that men had higher skin sodium than women after controlling for both age and BMI [44, 45, 48]. In cross-sectional studies on normotensives and hypertensives, Kopp et al. have shown that skin sodium was positively associated with BP, patients with refractory hypertension had increased tissue Na^+^ content compared to controls, and there was a trend for increased skin sodium in individuals with hyperaldosteronism [44, 45].

In 2014, Dahlmann et al. investigated the role of the skin as a sodium buffer using ^23^Na-MRI in 24 haemodialysis patients before and after a single dialysis. Skin sodium was reduced by 19% following dialysis, and patients with higher serum VEGF-C levels had better dialytic Na^+^ removal [46••]. Skin sodium was also higher in haemodialysis patients compared to healthy controls, and they noted an age-related rise in skin Na^+^ which corresponded to a decline in serum VEGF-C. The authors concluded that skin Na^+^ stores can be mobilised by haemodialysis, and VEGF-C facilitates Na^+^ flow between the interstitium and systemic circulation in humans, supporting earlier work in rodents [38, 46••]. More recently, Kopp et al. showed that type 2 diabetics on haemodialysis had significantly higher skin sodium compared with their non-diabetic counterparts [50••].

Schneider et al. used Na MRI to investigate the potential role of skin Na^+^ as a biomarker for hypertensive target-organ damage, further demonstrating that skin sodium content positively correlated with systolic BP and was a strong, independent predictor of left ventricular mass (LVM) in 89 participants with mild renal impairment [49••]. In these patients, skin sodium was independent of sex, height, SBP and body hydration as measured by bioimpedence.

In summary, cross-sectional data from ^23^Na-MRI studies in humans show that higher skin sodium storage is associated with higher BP and target organ damage. Skin sodium appears to be higher in older individuals, hypertensives, haemodialysis patients and diabetics—all groups previously known to have the SSBP trait. Skin sodium changes during dialysis support its role as a buffer for body sodium. VEGF-C appears to determine skin sodium in humans, potentially via lymphangiogenesis facilitating the efflux of sodium. Sex-specific differences in skin Na^+^ are interesting, but their relevance is currently unclear. Thus, it can be seen that ^23^Na MRI has provided important insights into the relevance of skin Na^+^ in humans.

## Insights from Direct Chemical Analysis of the Skin Sodium in Humans

Although MRI data were confirmed by direct ashing of human cadaveric samples, they have not yet been confirmed by direct chemical analysis of skin electrolytes in humans [44]. In the past year, two studies have evaluated skin electrolytes in humans using inductively coupled plasma optical emission spectrometry (ICP-OES), a highly sensitive analytical tool capable of simultaneous multi-elemental determinations down to the sub-parts-per-billion level [51, 52••, 53••]. Fischereder et al. measured tissue Na^+^ and GAG content in skin and arterial samples taken from renal transplant donors and recipients [52••]. They showed that skin and arterial Na^+^ concentration correlated with GAG content, suggesting that interstitial Na^+^ storage is regulated by GAGs in humans, and this could function in the buffering of dietary salt. They also found that skin Na^+^ correlated well with arterial Na^+^, indicating a possible link between the systemic vasculature and the skin with regard to sodium homeostasis.

We recently assessed skin electrolytes, blood pressure and plasma VEGF-C in 48 healthy participants (24 men) taking placebo (70 mmol sodium/day) and slow sodium (200 mmol/day) for 7 days in a double-blind, randomised, cross-over study [53••]. Skin Na^+^, expressed as the ratio Na^+^:K^+^, was 8% higher following the slow sodium phase. Post hoc analysis revealed a sex-specific effect, wherein men experienced a significant 11.2% increase in skin Na^+^:K^+^ following the slow sodium phase while women did not (4%). Women showed a significant increase in 24-h mean arterial blood pressure and body weight with salt loading while men did not. We concluded that skin sodium increases with dietary salt loading and this may be influenced by sex. Women showed a trend for less skin Na^+^ accumulation of salt loading and greater salt sensitivity of BP, in keeping with previous studies in rodents showing the skin functions as a buffer for dietary sodium [35–39]. We hypothesised that the sex differences observed could be due to sex-specific skin structural differences in thickness and GAG content and men having a greater capacity for passage of Na^+^ through the skin than women [54, 55]. In this study, skin Na^+^:K^+^ positively correlated with BP and peripheral vascular resistance (PVR), in support of recent ^23^Na MRI data showing a positive correlation between BP and skin Na^+^ [45, 49]. This was seen in men only, possibly due to variability in skin K^+^ and hence the Na^+^:K^+^ ratio with contraceptive treatment in women [53••]. No significant changes in plasma VEGF-C were observed between placebo and slow sodium phases to support a clear involvement of Ton-EBP or VEGF-C activation in this study.

## Potential Mechanisms Linking Skin Sodium and Blood Pressure

The exact basis for the relationship between skin sodium and BP is unknown. These parameters could be linked to a common yet unknown aetiological factor. Alternatively, skin sodium could mediate changes in haemodynamics either directly or through other substances. The Ton-EBP-VEGF-C axis has been shown to mediate BP in dietary salt loading. Several other mechanisms could explain the link between skin sodium and haemodynamics.

Skin capillary rarefaction, the reduction in the density of capillaries, and has been associated with hypertension [56–58]. Capillary rarefaction is believed to be structural in origin, associated with either impaired angiogenesis or capillary attrition and is believed to mediate BP changes by altering PVR [57]. He et al. showed that in hypertensive humans, a modest reduction in salt intake improves dermal capillary density as assessed by capillaroscopy [58]. This trend was seen across different racial groups and suggests that salt intake is linked to microvascular rarefaction. The exact mechanisms whereby salt affects the microcirculation remain unclear, with recent work in rats suggesting that skin sodium accumulation during high salt intake increases vasoreactivity in the skin [59].

The hypoxia inducible factor (HIF) transcription system, acting via the heterodimeric transcription factors HIF-1α and HIF-2α, plays a central role in the cellular response to hypoxia [60]. Recent evidence suggests that the HIF-1α:HIF-2α ratio in the skin affects synthesis of nitric oxide synthase 2(NOS 2), a key regulator of vascular tone [61]. HIF-1α and HIF-2a act antagonistically—HIF-1a promotes nitric oxide production by keratinocytes via NOS 2 while HIF-2α promotes keratinocyte arginase expression and urea production [62]. Cowburn et al. recently showed that mice with keratinocyte HIF-1α deletion had increased vascular tone and elevated systemic BP. Conversely, deletion of HIF-2α activity in keratinocytes resulted in increased skin NO levels and reduced systemic BP [61]. In accordance with this, they showed decreased epidermal expression of HIF-1α and increased epidermal HIF-2α expression in hypertensive humans correlated significantly with increased mean blood pressure. These findings provide a novel mechanism for systemic BP regulation by the skin. Recent evidence in rodents suggests that HIF metabolism may also be influenced by dietary salt. In the renal medulla dietary salt suppresses HIF prolyl-hydroxylase 2 (PHD2), which degrades HIF-1α and HIF-2α, increasing natriuresis [63, 64]. If high salt intake could similarly alter levels of HIF isomers in the skin, this would potentially influence PVR. This would need to be explored in further work.

## Movement of Dietary Sodium into the Skin—How and Why Does It Get There?

**Fig. 2:**
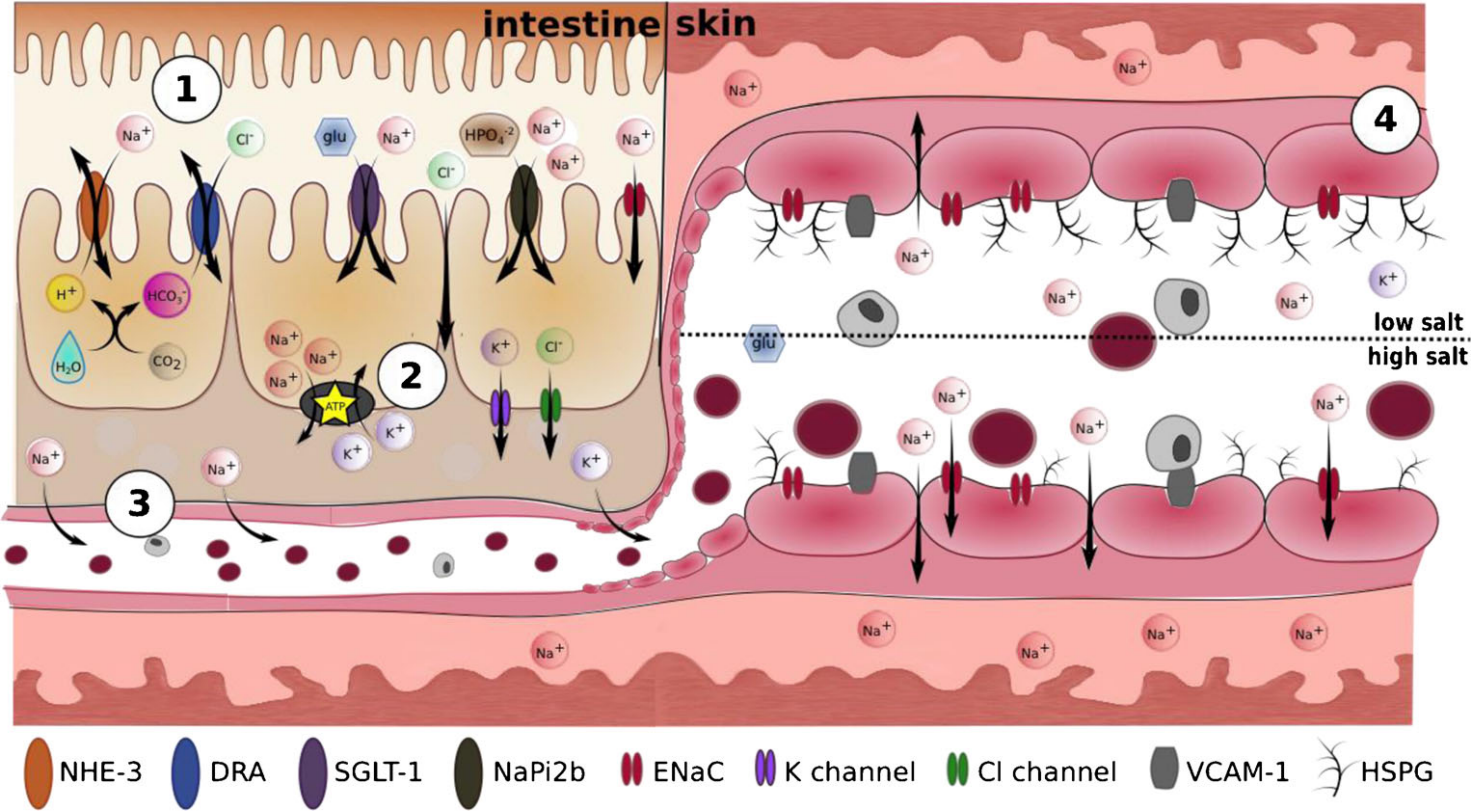
Movement of sodium from the intestinal lumen to the skin. [1] Intestinal sodium absorption across the apical membrane of enterocytes is facilitated by (i) Na-H exchange (NHE-2, NHE-3, NHE-8), (ii) cotransport with sugars and phosphates (SGLT-1, GLUT, NaPi2b) and (iii) diffusion through endothelial Na channels (ENaC). Chloride transport occurs via bicarbonate exchange (DRA) and paracellular diffusion. [2] Intracellular sodium is actively pumped across the basal membrane of the intestine by Na-K ATPases. [3] Once in the interstitium, sodium diffuses into the intestinal capillaries for transport through the vasculature. [4] Sodium can diffuse paracellularly into the skin under low salt conditions. Consuming excessive amounts of salt can exaggerate this process by causing damage to the endothelial glycocalyx and reducing barrier effectiveness

To reach the skin, dietary sodium must first transit through the intestine, the blood stream and then out into the dermal interstitium (Fig. 2). Sodium absorption across the apical membrane of enterocytes and colonocytes is broadly facilitated by three mechanisms: (1) sugar and phosphate co-transport via SGLT1, GLUT and NaPi2b; (2) electroneutral proton exchange via NHE-2, NHE-3, and NHE-8 and (3) passive diffusion via the sodium channel ENaC [65–69]. This latter mechanism is predominantly localised to the colon.

In contrast, chloride absorption is largely mediated by paracellular diffusion and apical bicarbonate exchange via downregulated-in-adenoma (DRA) proteins. This bicarbonate exchange is coupled to NHE-mediated sodium absorption, effectively marrying Na^+^/H^+^ and Cl^−^/HCO_3_^−^ exchange [70]. Intracellular chloride can diffuse out of enterocytes through chloride channels.

Intracellular sodium is actively pumped across the basolateral membrane and into the extracellular space by sodium-potassium ATPases, which export three sodium ions from the cell in exchange for two potassium ions. The requisite extracellular potassium concentrations are maintained by diffusion of intracellular potassium through basolateral potassium channels. After concentration in the extracellular space, sodium can diffuse into the intestinal capillaries for transport throughout the body. It is not clear whether these various sodium, chloride and potassium transporters play a role in sodium sensitivity.

What drives sodium out of vasculature and into the skin specifically is not well understood, and the transit route from vascular lumen to dermis is likewise speculative. When transitioning out of the vascular lumen, sodium first encounters the endothelial glycocalyx. Comprised primarily of heparan sulphate proteoglycans (HSPGs), this delicate layer varies in thickness from 0.5 to 4.5 μm [71]. The anionic character of this glycocalyx facilitates smooth red blood cell movement, inhibits white blood cell adhesion, scavenges oxygen free radicals and can help detect small changes in blood pressure and trigger a vasodilatory response [71, 72].

Excessive dietary sodium consumption may damage this glycocalyx and promote sodium ‘leakage’. In vitro experiments have shown that chronic exposure of endothelial cells to 150 mM sodium decreased glycocalyx HSPGs by 68% and caused endothelial stiffening [73, 74]. This damaged glycocalyx could facilitate excessive sodium movement into the interstitium via paracellular diffusion and increased exposure to or increased activation of vascular ENaC channels [75]. Additionally, glycocalyx damage and endothelial stiffening may increase leucocyte adhesion and infiltration, further damaging the vessel wall [73, 75, 76].

From current data, it is not immediately clear whether the skin acts as a pre-emptive reservoir for excessive sodium, removing it from circulation before it can induce adverse cardiovascular effects, or if the skin functions as an overflow reservoir once the excessive sodium has caused sufficient vascular damage to ‘leak’ into the surrounding tissue. Regardless, once in the skin, the positively charged sodium is osmotically inactivated through association with anionic GAGs. The presence of high concentrations of sodium can stimulate increased GAG synthesis, expanding the storage capacity of this osmotically inactive third compartment.

## Conclusions and Future Directions

The skin acts as a third compartment for sodium, capable of non-osmotic sodium storage and mediating a vasodilatory response via VEGF-C. This appears to constitute an extra-renal mechanism controlling blood pressure during high salt intake. Incorporating this model into the traditional paradigm of sodium homeostasis, the skin may act as a buffer as well as a reservoir for sodium, while the kidney controls sodium excretion and reabsorption, controlling serum osmolality and total body water. It is conceivable that people predisposed to salt-sensitive hypertension have defects in the pathways described above. What is less understood is why skin sodium accumulation with short-term dietary salt loading appears to protect from a rise in BP rise, but long-term high skin sodium is associated with higher BP and the propensity for SSBP. A potential explanation could be that impaired VEGF-C-induced lymphangiogenesis in these individuals reduces efflux of sodium from the skin to the systemic circulation and attenuates the vasodilatory response to salt loading. Further work is needed to explore this and other mechanisms that could be involved. The exact mechanisms underlying the movement of dietary sodium from the gut to the vasculature and the skin are yet unclear. Future challenges include ascertaining how different antihypertensive agents affect the distribution of sodium and water between the skin and the intravascular space and how this system interacts with other organs that modulate BP like the kidney and brain.
